# The proposed hybrid deep learning intrusion prediction IoT (HDLIP-IoT) framework

**DOI:** 10.1371/journal.pone.0271436

**Published:** 2022-07-29

**Authors:** Magdy M. Fadel, Sally M. El-Ghamrawy, Amr M. T. Ali-Eldin, Mohammed K. Hassan, Ali I. El-Desoky

**Affiliations:** 1 Computer Engineering and Systems Department, Faculty of Engineering, Mansoura University, Mansoura, Dakahlia, Egypt; 2 Head of Communications and Computer Engineering Department, MISR Higher Institute for Engineering and Technology, Mansoura, Dakahlia, Egypt; 3 Mechatronics Department, Faculty of Engineering, Horus University in Egypt (HUE), New Damietta, Damietta, Egypt; Hanyang University, REPUBLIC OF KOREA

## Abstract

Throughout the past few years, the Internet of Things (IoT) has grown in popularity because of its ease of use and flexibility. Cyber criminals are interested in IoT because it offers a variety of benefits for users, but it still poses many types of threats. The most common form of attack against IoT is Distributed Denial of Service (DDoS). The growth of preventive processes against DDoS attacks has prompted IoT professionals and security experts to focus on this topic. Due to the increasing prevalence of DDoS attacks, some methods for distinguishing different types of DDoS attacks based on individual network features have become hard to implement. Additionally, monitoring traffic pattern changes and detecting DDoS attacks with accuracy are urgent and necessary. In this paper, using Modified Whale Optimization Algorithm (MWOA) feature extraction and Hybrid Long Short Term Memory (LSTM), shown that DDoS attack detection methods can be developed and tested on various datasets. The MWOA technique, which is used to optimize the weights of the LSTM neural network to reduce prediction errors in the hybrid LSTM algorithm, is used. Additionally, MWOA can optimally extract IP packet features and identify DDoS attacks with the support of MWOA-LSTM model. The proposed MWOA-LSTM framework outperforms standard support vector machines (SVM) and Genetic Algorithm (GA) as well as standard methods for detecting attacks based on precision, recall and accuracy measurements.

## 1 Introduction

In the huge IoT network, where digital devices are interconnected, security is a real necessity. It is crucial that device-to-device communication is cooperative and ensures data security. As more devices are connected to the network, the level of confidentiality decreases. There is a tremendous increase in the effects of overcoming this challenge. In [1, 2] IoT devices contain sensitive data that is more vulnerable to simple attacks, which leads to a large number of compromised devices. IoT networks are diverse, thus accounting for these differences. Providing the new device with auto-configuration will pose a security risk. IoT devices are manufactured without considering standards, because the manufacturers are in a hurry to release their products [3].

Security holes can be found at different stages of design using different scanning tools. Recent research proposed using a stresser and booster to detect DDoS attacks. It is difficult to distinguish between DDoS and flash crowd because they vary with minimum parameters [4]. Flash crowd triggers the attack based on traffic intensity. In [5] by applying Box-Cox transformation to the packet time series, it can be used to make better predictions based on some of the characteristics of attack time. A centralized interface is provided in [6] which constantly check the current level of infection and the current attack device. DDoS poses a significant threat because it attacks and floods distributed computers, blocking the server or communication channel. An innovative solution, HDLC, is proposed in this paper as a meritorious way to defend against these threats.

The proposed deep learning framework utilizes an optimized LSTM Neural Network (NN) with a set of optimally extracted features to classify and detect new offensive packets. In conjunction with both of these methods, the network can automatically learn new attack patterns and append them to a database of attack signatures [7].

The IoT environment has recently attracted attention due to its potential use in a variety of human activities. Since sensor prices have fallen, solutions that improve the quality of people’s lives have become more popular. Using IoT devices, resources can communicate easily, with such ease of access comes a price: a need for security. The level of confidence in data obtained from IoT devices is another concern, and how or where such data can be used is one of the motivations for such research [8].

Software defined networks (SDN) are more suitable for huge networks due to their centralized management [9, 10], dynamic and programmable architecture. Generally, Distributed Denial of Service (DDoS) attacks involve bombarding a network with a large number of packets so as to hinder or even prevent legitimate users from reaching the network [11, 12]. SDN attacks target only the network’s controller, whereas traditional networks have many points of attack [13]. Furthermore, the attacking packets often pose as having fake destination IP addresses, however in traditional networks, the destination IP address should actually be the IP address of the targeted server [13]. Detection and defense techniques for SDN are basically an imitation of traditional network techniques, without accounting for SDN’s own characteristics. Attack detection and defense are implemented on the SDN controller, increasing the computational overhead on the processor, as well as the communication between the controller and switches (southbound) [13]. A variety of meta heuristic optimization techniques have been applied to overcome these problems, including Genetic Algorithm (GA) [14], Particle Swarm Optimization (PSO) [15], Firefly Algorithm (FF) [16], and Modified Whale Optimization Algorithm (MWOA) [17]. There are many limitations and complexity associated with them; such GA is dependent on the initial population and may be unable to achieve convergence in the parameters [14]. Further, PSO lacks a good control on discrete optimization problems and falls into local optima easily [15]. Because of these limitations, many hybridized and improved versions of Machine Learning (ML) have been developed.

To reduce both processing and communication overhead, this paper detects suspicious traffic at the data plane (switches). To improve the accuracy and speed of the classification process, two techniques based on signatures [11, 18] and deep learning are employed to classify suspicious packets executed at the controller plane. Signature-based systems basically use intermediate routers to compose unique patterns (signature) for every packet passing through the network [19]. An attack signature database stores the signatures of malicious packets, allowing them to be matched later against any attack packet containing a signature. A high degree of accuracy and low false-negative rates can be achieved with this technique, but it is unable to detect new (day-zero) attacks that are not included in the attack signatures database [20].

An optimized deep learning framework is proposed that uses an optimized LSTM-NN to classify and detect new offensive packets based on a set of features extracted from a number of original set. With both techniques combined, the network is able to learn new attack patterns then add them automatically to its attack signature database for later use [21].

DDoS attacks are the subject of numerous studies at present. It is important to know the advantages and disadvantages of DDoS attacks when designing an architecture able to predict them. Among the studies, it became clear that DDoS attacks have a structure that is very similar to other types of attacks, leading to an incorrect classification of these attacks. It is therefore extremely important to select an appropriate classification technique. To overcome these deficiencies, in the present paper, the prediction of DDoS attacks within the environment of IoT is performed using a deep learning technique such as MWOA as part of a LSTM structure.

## 2 Motivations and contribution of this paper

This paper’s motivation is as follows:

1. Provide a framework that copes with big data and sharply classify network traffic more effectively using DL H2O Binary MWOA.
2. Utilize a modified version of MWOA for Feature Selection (FS) to reduce the data set size and also to tune (select the ideal number of layers and neurons/layer) the LSTM neural network parameters’.

This paper contributes the following:

- A DL framework that supports a wide variety of traffic data formats, since IoT devices contain multisource formats. It can also be used with SDN environments.
- An attack detection system that relies on deep learning and signatures is being developed in order to enhance detection accuracy and processing speed.
- FS helps in selecting optimal features from the input data when training a NN; this reduces the input size to LSTM, and thus improves traffic type prediction.
- It has been evaluated on three datasets including NSL-KDD [22], CSE-CIC2018 [23], which are the most traditional network’s datasets commonly used, and a realistic virtual SDN network created by Mininet emulator [24] to show its reliability, and these data have been used in most current studies.

As a continuation of this paper, the next section gives an overview of related work, section 4 details the proposed framework, section 5 presents experimental results and evaluations of performances, and ultimately section 6 concludes the study.

## 3 Related works

In this section, we review works that make use of entropy and machine learning based algorithms to handle security issues in IoT-SDN environments.

### 3.1 Entropy detection approaches

Entropy approaches are also called statistical approaches since entropy measures how random a dataset is. Since each feature in normal network traffic is distributed in a certain pattern, for example, balancing between the number of source and destination IP addresses. Due to the extensive increase in destination IP addresses relative to source IP addresses, the entropy balance in attack flows will migrate. Nevertheless, this approach is characterized by its rapid response time and low computation overhead when a significant amount of traffic data is processed. Correct selection of the threshold value for a specific traffic feature can greatly improve the detection accuracy and reduce the rate of false positives and false negatives.

Using entropy and traffic volume characteristics together, authors propose in [25] an improved attack detection system that offers better results than using either technique alone. In the study by Kalkan et al. [26], a joint entropy-based scoring system (JESS) was introduced to protect SDN environments, allowing them to combat even unknown attacks. Using a statistical approach to traffic entropy in SDN environments, Lima et al. [27] proposed a new system. Using entropy measurements of flow data to detect attacks and improve the CPU’s utilization and dynamic response to attacks, a novel solution has been developed in [28].

According to Ahmed et al [29], a new structure called application fingerprints are used to identify legitimate packets from attack packets by expressing packet attributes and flow statistics. As a consequence, this approach is not suitable for online systems, as some attributes of a flow, such as total bytes, number of packets between sources and destinations; flow duration and flow duration as a function of direction aren’t able to be calculated. [30] Presented new hybrid approaches that combined flow level statistics and entropy based methods with some techniques of Deep Learning (DL) or Artificial Neural Networks (ANN) to resolve some deficiencies associated with flow statistics. DL and ANN are detailed in the next sub-section.

### 3.2 Deep learning (DL) prediction approaches

Recent years have seen machine learning play an increasingly important role in assisting with IoT security and detection [31, 32]. Recent years have also seen considerable interest in deep learning. Presently, this method of intrusion prediction in networks is recognized as a relevant one.

A recent survey by Conti et al. [33] examines the challenges and opportunities of the IoT domain. As portrayed by the authors, it is imperative that a successful IoT network can identify compromised nodes, collect evidence of attacks, and preserve evidence of a malicious attack. Study primarily aimed at outlining significant challenges associated with IoT. According to the authors, IoT systems are positioned passively and autonomously by design, so detecting their presence is a challenge.

In [34], Diro and Chilamkurti present new techniques for intrusion detection in the IoT context based on deep learning with promising results. Additionally, the authors report that, because of the addition of various protocols, mainly from IoT, thousands of zero-day attacks have also been discovered. Many of these attacks are minor variations of cyber-attacks previously reported. In such cases, detecting these small mutants of attacks over time is difficult, even using traditional machine learning systems.

Lopez-Martin et al. [35] developed a new method for identifying intrusions in IoT networks. It uses a Conditional Variational Autoencoder (CVAE) based on an architecture that integrates intrusion labels within the decoding layers. In addition to the capability of re-constructing features, the proposed model may also be used in systems such as the Network Intrusion Detection System, which is part of network monitoring systems, and in particular IoT networks. By using a single training step, the proposed approach reduces computational requirements.

Regardless of the fact that IoT will be a future part of 5G networks, Fu et al. [36] argue that it is not without its limitations because the safety of the future 5G network is difficult to implement many security mechanisms on IoT. A new approach to handling vast heterogeneous IoT networks is presented based on the automata theory. To detect the intrusion, the method uses an extension of Labelled Transition Systems to describe IoT systems uniformly. The descriptions are based on the comparison of action flows.

The IoT poses implicit challenges and is much more complex than the conventional approach to privacy and intrusion detection, as Gunupudi and colleagues [37] showed. In this work, the goal is to represent each high dimensional sample in the global dataset by a method equivalent to a reduction in dimension, using a membership function to cluster attributes incrementally. A dimensionality reduction approach was used to obtain a reduced representation that was used for training classifiers.

A security architecture based on software-defined networking (SDN) for Internet of Things was discussed by Flauzac et al. [38]. In this context, SDN-based architecture can function with or without infrastructure, otherwise known as SDN-Domain. The work describes the proposed architecture in detail and discusses the possible use of SDN for increasing network security efficiency and flexibility. Several architectural design choices for SDN utilizing OpenFlow and their performance implications were discussed in this article, including network access control and global traffic monitoring for ad-hoc networks.

According to Cruz et al. [39], there is a need for an Internet of Things middleware because the devices tend to have limited resources. The use of such middleware could enable the processing of intelligently based decision-making mechanisms.

## 4 The proposed hybrid deep learning intrusion prediction IoT (HDLIP-IoT) framework

**Fig 1:**
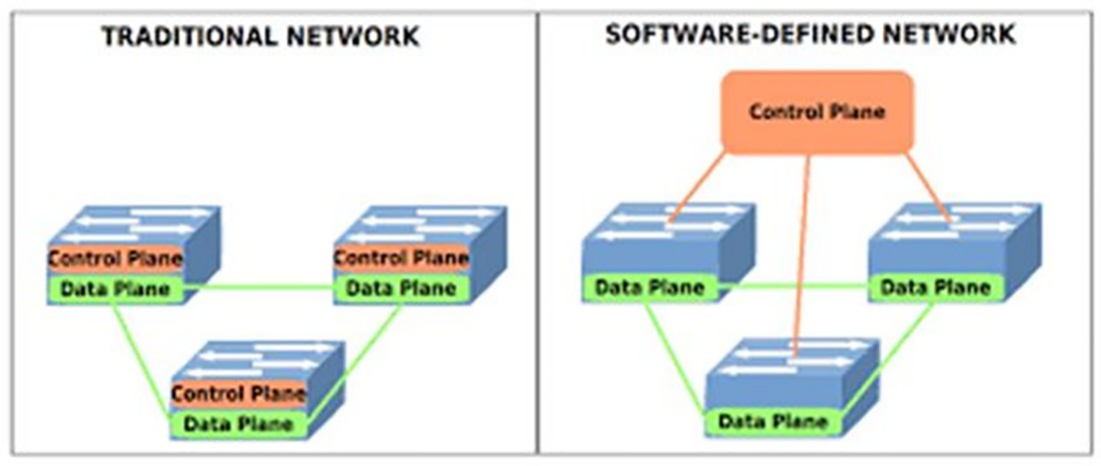
Traditional versus SDN network architectures.

Due to the customary duties assigned to traditional networks’ routers, such as determining packet routes and priority, carrying out administrator polices, and many other things, they are unable to detect and respond to DDoS attacks automatically. SDN-based architecture provides a fast and accurate response to these attacks by automatically handling them. Fig 1 illustrates this.

**Fig 2:**
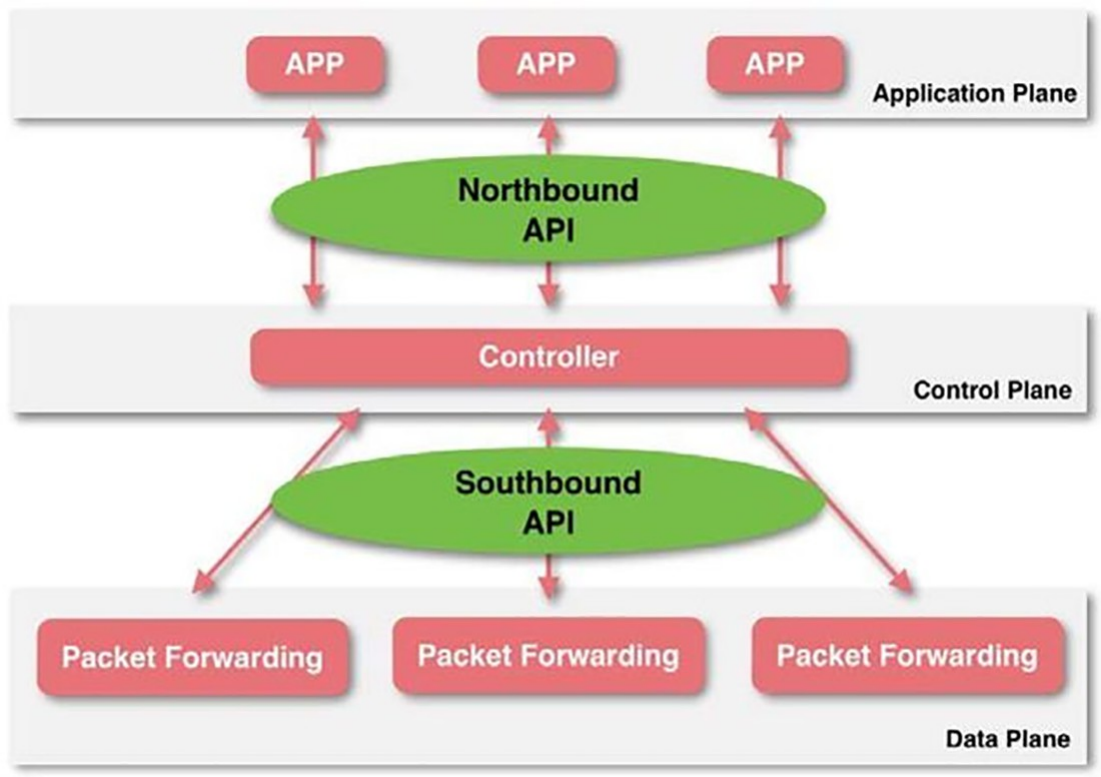
SDN three layers architecture.

Fig 2 illustrates a three-layer SDN architecture, where the switching function is split between a data layer and a control layer implemented on separate devices. Data planes are primarily responsible for forwarding network packets, while the control plane is responsible for performing all intelligence operations on the network. According to standard SDN, the control layer is responsible for both attack detection and defense, so it may require heavy CPU use and high communication workload. This will increase the controller’s workload and CPU utilization. It will monitor hourly the traffic going through switches to identify DDoS attacks.

**Fig 3:**
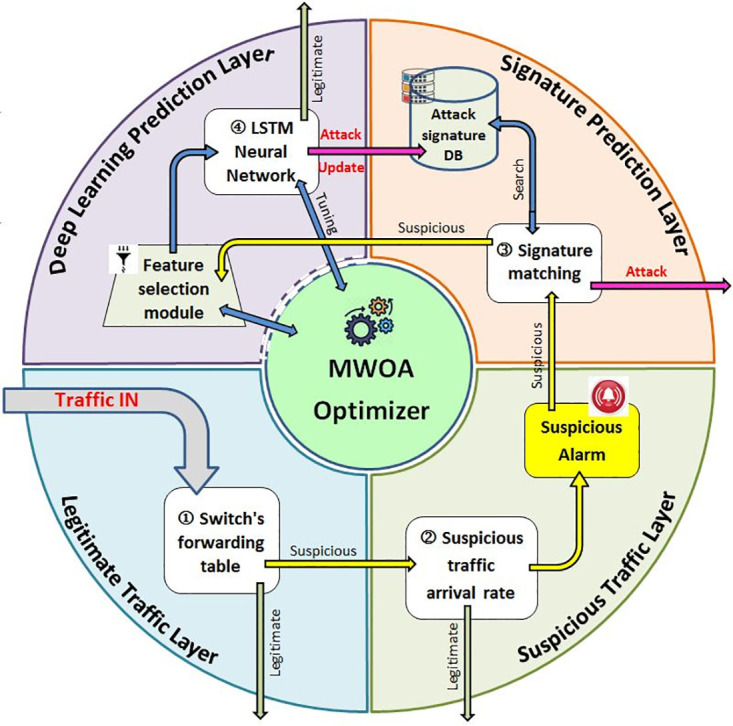
The proposed (HDLIP-IoT) framework.

The proposed framework (HDLIP-IoT) may help to overcome this defect. It is made up of four layers, Legitimate Traffic Layer, Suspicious Traffic Layer, Signature Prediction Layer and Deep Learning Prediction Layer as depicted in Fig 3.

### 4.1 Traffic detection layers

The lower two detection layers determine any suspicious flow and raise an alarm to the upper two prediction layers to determine if it is a DDoS attack or just a valid flash crowd since they are varying in minimum parameters before taking corrective action. Therefore, it would be possible to reduce traffic overload through the Southbound interface and thereby decrease CPU controller workload. Here are the two layers:

#### 4.1.1 Legitimate traffic layer

SDN packets arrive first at the data plane, they have four possible classifications: Known traffic which has already been entered into the switch’s forwarding table; thus, it is redirected to its proper destination. The second type of legitimate traffic is a new valid route that the switch cannot first find in its forwarding table and will receive a reply from the controller with a suitable forwarding route that will be added to its forwarding table in future. The other two categories occur when the switch receives a suspicious packet. An IP address tampering in the source or destination addresses of the packet prevents the controller from determining its routing route, since it is not found in the forwarding table. It is important to consider the arrival rate of the two last categories of packets (suspicious) when managing DDoS detection.

#### 4.1.2 Suspicious traffic layer

By using the maximum packet counter method at the data plane, the arrival rate of suspicious packets is determined within a predefined window of time. In the framework, the suspicious flow counter (Susp++) is incremented when a switch classifies a packet as a suspicious flow and its features are added to both the training dataset and the current interval dataset. Following that, it compares the value of Susp to an adaptive maximum attacking packet value (Val). When Susp is less than Val, the detection is safe, so drop this packet and process any incoming packets.

Alternatively, if Susp is greater than or equal to the predefined value (Val), the detection system calculates the time window, packet arrival rate (PR), and initializes all counters. The control layer receives a suspicious alarm when the packet arrival rate (PR) exceeds the predefined value.

### 4.2 Traffic prediction layers

Signature prediction and Deep Learning prediction are two sub-layers of this layer.

#### 4.2.1 Signature prediction layer

Routers that support traceback insert their identification IDs into packet headers by using one of two methods, Deterministic Packet Marking (DPM) [40] or Probabilistic Packet Marking (PPM) [41]. Path signatures are formed as the result of collecting all identifiers along the packet’s path. The exact route of a packet is determined by this indicator regardless of the source IP address which may be forged. The attack signature database stores the signature of the isolated attacking traffic for future reference after it has been isolated.

#### 4.2.2 Deep learning prediction layer

This final stage of the classification process identifies the fourth category of suspicious packets. Based on the large exploration of the search space, simplicity of implementation, wide range of applications, and development potential of the Modified Whale Optimization Algorithm (MWOA) [17], we can choose the optimal set of features and tune parameters of the classification NN.

MWOA is a mathematical algorithm that simulates the hunting mechanism of humpback whales. In the exploration phase, whales search for prey in random directions as they like to hunt together, which is illustrated in this model.

$$\vec{D}=\left|\vec{C}{\vec{.X}}_{rand}-\;\vec{X}\right|$$ (1)

$$\vec{X}\left(t+1\right)=\;{\vec{X}}_{rand}-\;\vec{A}\vec{.D}$$ (2)

Where: ${\vec{\text{X}}}_{\text{rand}}$ is a random position vector (random whale) and t indicates the current iteration.

Due to the fact that the best position of the prey in the search space was unknown previously, the whales communicated to determine what the best solution was. In the next step, which is called the exploitation phase, other whales will update their directions toward the current elected whale, modeled as follows:

$$\vec{\text{X}}\left(\text{t}+1\right)=\;\vec{\text{D}\prime }{\text{e}}^{\text{bt}}\;\text{cos}\left(2\pi \text{t}\right)+\;\vec{\text{X}*}\left(\text{t}\right)$$ (3)

Where: $\vec{\text{D'}}=\;\left|\vec{\text{X}*}\left(\text{t}\right)-\;\vec{\text{X}}\left(\text{t}\right)\right|$ indicating the i^th^ of whale the prey (best solution obtained so far), b is constant defining the shape of legitimate spiral and t is a random number in [-1, 1].

$$\vec{\text{X}}\left(\text{t}+1\right)=\left\{\begin{array}{ll} \vec{\text{X}*}\left(\text{t}\right)-\vec{\text{A}}.\vec{\text{D}}\;, & \text{p}<0.5\\ \vec{\text{D}\prime }\;{\text{e}}^{\text{bt}}\;\text{cos}\left(2\pi \text{t}\right)+\vec{\text{X}*}\left(\text{t}\right), & \text{p}\geq 0.5 \end{array}\right.$$ (4)

Where: p is a random number in [0, 1].

Within an encircling prey, a search agent will attempt to update its position in relation to the best search agent after identifying the best search agent, following equations illustrate this behavior:

$$\vec{\text{D}}=\left|\vec{\text{C}}\;.\;{\vec{\text{X}}}_{\text{P}}\left(\text{t}\right)-\vec{\text{X}}\left(\text{t}\right)\right|$$ (5)

$$\vec{\text{X}}\left(\text{t}+1\right)={\vec{\text{X}}}_{\text{P}}\left(\text{t}\right)-\vec{\text{A}}.\vec{\text{D}}$$ (6)

Where: ${\vec{\text{X}}}_{\text{P}}$ is the position vector of the prey, $\vec{\text{X}}$ is the position vector of a whale and $\vec{\text{A}}$ and $\vec{\text{C}}$ are coefficient vectors.

Feature reduction will involve an extensive search for reducing N features in the dataset. To attain the optimal feature combination, the proposed algorithm was used to adopt the search space. Furthermore, the fewer the chosen features, the better the solution. Every solution was measured using a special fitness function; the function is based on two primary metrics: the number of features selected in the solution (L) and the error rate (E_R_(D)). These objectives were achieved by utilizing an NN. Additionally, using our fitness function, which is defined as follows, these features were used to train the NN for achieving the best model:

$$Fitness=\;\alpha E_{R}\left(D\right)+\beta \frac{\left|L\right|}{\left|N\right|}$$ (7)

**Fig 4:**
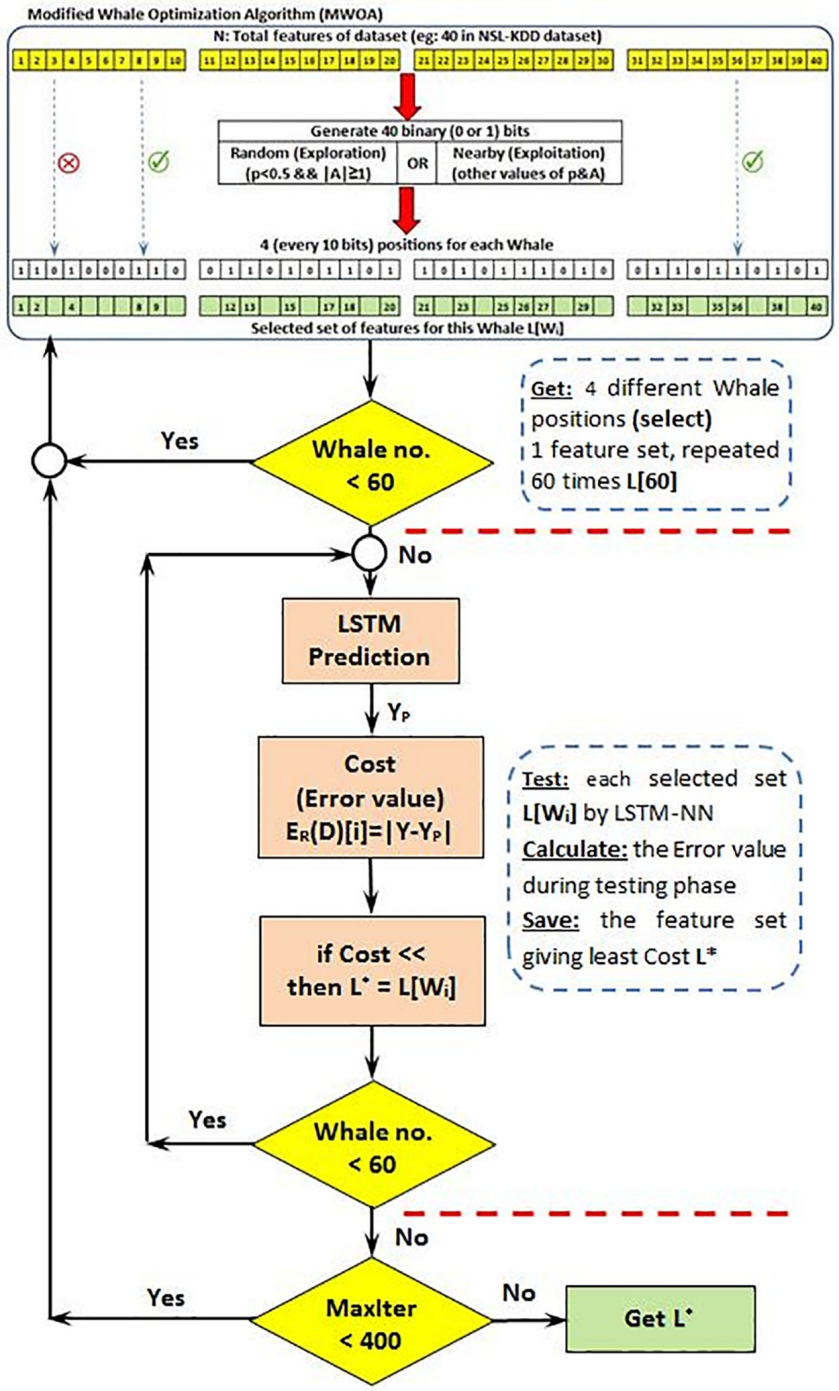
MWOA selecting feature block diagram.

The pseudo of MWOA is shown in Algorithm 1 and Fig 4.

**Algorithm 1. Pseudo code of selecting optimal set of traffic features using MWOA and LSTM**.

**Input**: All of the features relating to network traffic

**Output**: An optimal combination of features

The parameters of the algorithm (SearchAgentsNo = 60, dim = 4, LB = 0, UB = 1023 and MaxIter = 400) are initialized as shown in Table 1.

Initiate the MWOA relevant parameters (a, A, C, I, p and the positions L of whales)

1: Every Whale has 4 random position values each of 10 bits (ranging from LB to UB), and each is the selected set of 40 features from network traffic

2: StartTime = Time()

3: The LSTM network with its input as the whale position (or a set of selected features) is used to calculate each individual whale’s error value (cost).

4: **while (t < MaxIter)**

5:  **for each Whale (from 60 Whale)**

6:   Update a, A, C, I and p

7:   **if (p < 0.5)**

8:    **if (|A| < 1)**

9:     The current Whale’s position is updated by Eq (6) (encircling prey)

10:    **else (|A| ≥ 1)**

11:     Select a random search agent (L_rand)

12:     The current Whale’s position is updated by Eq (2) (exploration phase)

13:    **end if**

14:   **else (p ≥ 0.5)**

15:    The current Whale’s position is updated by Eq (3) (exploitation phase)

16:   **end if**

17:  **end for**

18:  Ensure that any Whale has a position (features value > 40) that exceeds the LB or UB and amend it if needed

19:  Calculate the Cost of each Whale using the LSTM neural network

20:  Updating L* (the set of features that gives the lowest error value or cost)

21:  t = t + 1

22: **end while**

23: Execution Time = StartTime − Time()

24: return L*

**Table 1:** Configuration values for the WOA optimizer.

Configuration	Value
MaxIter	400
SearchAgentsNo	60
Dimension	No. of features in the dataset
No. of run repetitions	15
*α* Parameter in the fitness function	0.99
*β* Parameter in the fitness function	0.01

**Fig 5:**
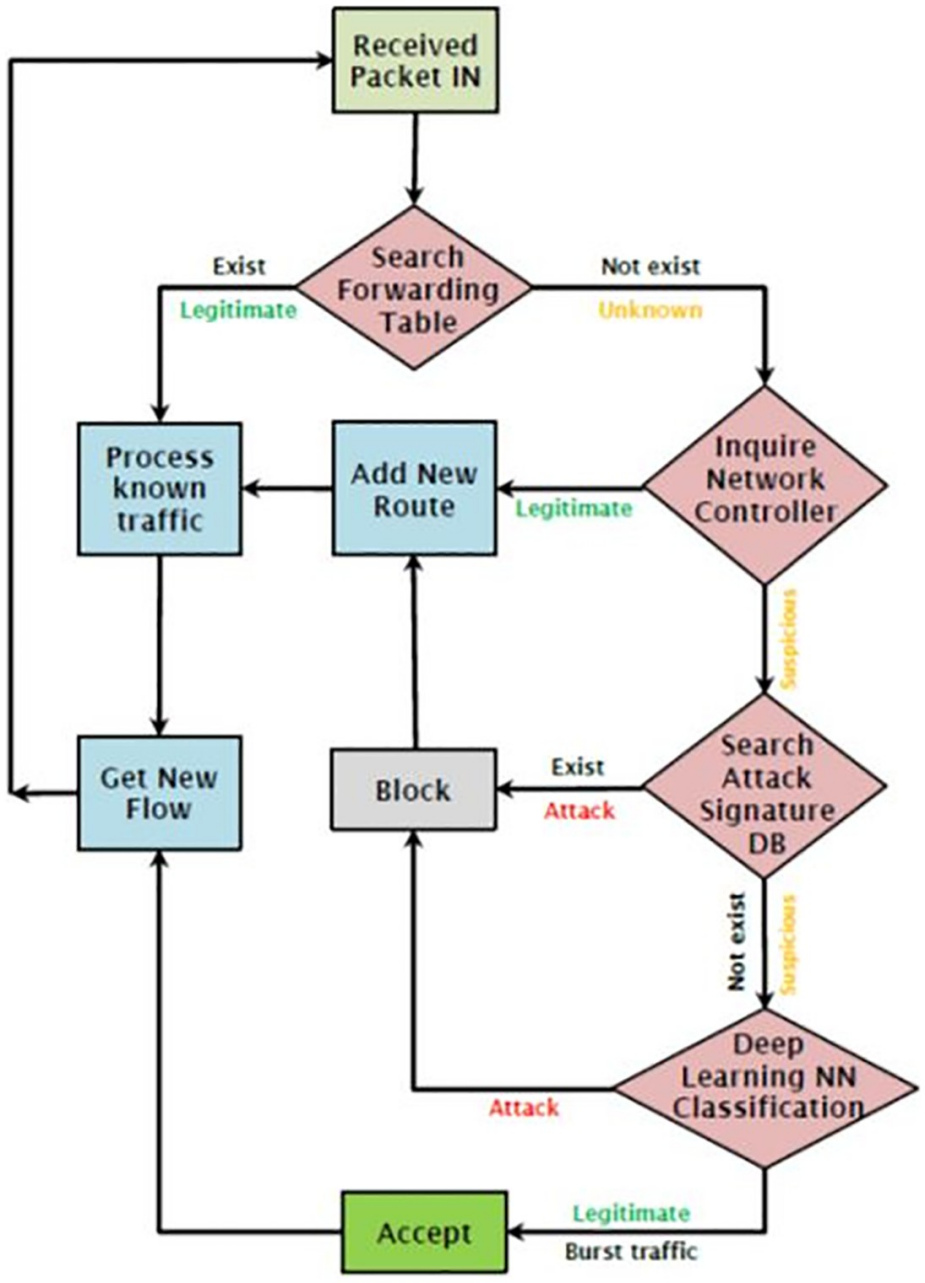
Received packet flow status.

Fig 5 represents the four stages in which the status of a packet is determined. An SDN environment can basically perform the first two stages (searching the switch’s forwarding table and asking the controller for the packet forwarding path). Additionally, the two last stages (searching a DB of attack signatures and deep learning) are appended.

LSTM networks can identify repeating attack patterns inside long packet sequences as opposed to traditional Machine Learning (ML) techniques. LSTM has emerged as an RNN model intended to solve RNN model problems, such as the internal status of RNN networks that reveal dynamic subsequent behaviors. As opposed to neural networks, the RNN uses its internal memory to store arbitrary time series input pieces to enable the processing of this type of model. The vanishing gradient and explosive gradient are also problems in RNN. LSTM was originally designed for the purpose of resolving long-term RNN dependence, because retrieving long-term data is not fundamentally a matter of learning, but rather a matter of standard neural network behavior. In the LSTM model, LSTM cells are replaced by RNN layer cells for long-term memory. LSTM models use both forgotten gates as well as input gates for introducing parameters. The forward calculation method can be demonstrated in the LSTM neural network as described in [42].

Algorithm 2 and the logic diagram of the received packet flow status in Fig 5 clarify the classification process in detail.

**Algorithm 2. The pseudo code of the proposed framework after tuning the LSTM-Neural Network**.

**Input**: Packet received at network switch.

**Output**: Identifying packet types for correct routing.

1: **Stage 1: Search Forwarding Table**:

2: Check the forwarding table of the Switch for the packet.

**3: If** (exist)

4:  A legitimate and old packet

5:  Direct it to the proper recipient

6:  Get a new packet to process

7: **Else**

8:  New packet

9:  **Inquire**: the network controller (Stage 2:) to identify its route (if possible)

10: **End if**

11: **End Stage 1**:

12:

13: **Stage 2: Inquire Network Controller**:

14: Identify the new packet’s route (if possible)

15: **If** (route found)

16:  New and normal packet

17:  Send its route to the inquiring switch

18:  Adding this route to the forwarding table of the switch for later use

19:  Forward the received packet

20:  Get a new packet to process

21: **Else**

22:  Suspicious packet alarm

23:  **Search**: Attack signature DB (Stage 3:) to determine if it is an attack or suspicious

24: **End if**

25: **End Stage 2**:

26:

27: **Stage 3: Search attack signature DB**:

28: In the signature database, search for the packet’s signature

29: **If** (signature exist)

30:  Old and attack packet

31:  Send it back to the inquiring switch to:

32:  • Block it

33:  • Add it to the forwarding table for future use

34:  Get a new packet to process

35: **Else**

36:  Suspicious packet trigger

37:  Conduct the LSTM NN classifier (Stage 4:) to determine if it is an attack or valid flash crowd

38: **End if**

39: **End Stage 3**:

40:

41: **Stage 4: Deep Learning NN Classifier**:

42: Classify the new packet

43: **If** (legitimate burst traffic)

44:  New and legitimate packet

45: **Else**

46:  New but attack packet

47: **End if**

48: Send it to the inquiring switch

49: The switch adds its state (attack or legitimate) to the forwarding table for future use

50: Forward it if a valid packet

51: Receive a new packet of data to process

52: **End Stage 4**:

## 5 Experiments and evaluation

A hybrid classification algorithm composed of MWOA and a tuned LSTM Neural Network in Tables 1 and 2 is used in experiments. MWOA is used to select the most effective set of features from the used datasets, whereas the tuned NN is used to accurately classify the newly unknown suspicious packets, reducing the computation overhead and increasing the IDS classification accuracy.

**Table 2:** Parameters of the NN structure.

Structure Parameters	Value
No. of hidden layers	2
No. of neurons	10
Biases	Random
Activation function	TanH
Initial weights	Default

### 5.1 Benchmark datasets

The framework is evaluated using three datasets shown in Table 3. Despite its simplicity, NSL-KDD dataset isn’t the best representation of any real network model; however CIC-IDS2018 dataset represents real attacks and makes an accurate evaluation of any IDS possible.

**Table 3:** A list of datasets used for the evaluation in the paper.

No.	Dataset	No. of features	No. of records	No. of classes	
				Normal	Attack
1	NSL-KDD	41	4,898,430	1 sub-type	Feature no. 42
				2,596,168 record	4 sub-types
					2,302,262 record
2	CIC-IDS 2018	83	3,119,345	1 sub-type	Feature no. 84
				2,359,087 record	6 sub-types
					760,258 record
3	SDN	23	623,869	3 sub-type	Feature no. 24
				471,818 record	3 sub-type
					152,051 record

The CIC-IDS2018 dataset has some drawbacks, mainly the enormous processing and load time required because of its huge number of records. Secondly, it is missing some data. Class imbalance [43] is the last demerit. So, this dataset will make the classifiers bias toward the majority class [43], which will reduce the accuracy of the classifier because the false rate will be higher.

As traditional networks are designed differently from SDNs, the data they gather is also very different from that which is gathered from SDNs. A realistic virtual network has been created with the help of the Mininet emulator [24].

### 5.2 Data preprocessing

Network collected dataset may include some error values such duplicate or categorical data, this may cause classification problems. These error values should be treated before training and testing phase. Duplicate records should be removed; techniques like one-hot encoder are used to convert categorical data into numeric values.

Feature scaling methods (Normalization and Standardization) [44]: features having values of varying degrees of magnitude, may hurdle the performance of some machine learning algorithms especially those types using gradient descent as optimization techniques.

Data imbalance reduction [43]: where some features are highly underrepresented, causing the classifier to bias towards the majority features. Many techniques are designed to handle class imbalance problem, one of them deployed in this paper is class relabeling. By either splitting the majority classes into more classes or merging some minority classes to form one class.

### 5.3 Evaluation metrics

**Fig 6:**
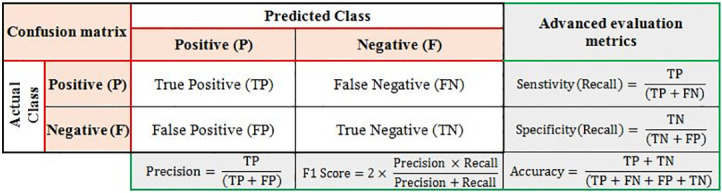
Confusion matrix and evaluation metrics.

Evaluating the trained model’s performance can be done using the confusion matrix and advanced evaluation metrics are shown in Fig 6.

True Positive (TP) represents the number of positives that are correctly judged as positive. False Negatives (FN) refer to the number of positive classes mistakenly classified as negatives. False Positives (FP): indicates the number of negatively classified individuals mistakenly labeled as positively. And finally, True Negative (TN): indicates the number of negative classes that were actually ruled negative.

### 5.4 Experimental results

Three experiments have been conducted to validate the performance of the (HDLIP-IoT) framework.

**Experiment 1: (NSL-KDD dataset)**: using a two hidden layers tuned LSTM-NN classifier.

Table 4 shows the advanced metrics obtained from the confusion matrix in Table 5. The average results are 98.379, 92.628, 98.657 and 95.469 for Accuracy, Precision, Recall and F1 Score, respectively.

**Table 4:** NSL-KDD metrics of the above confusion matrix.

No.	New Labels	Confusion matrix metrics				Advanced evaluation metrics (%)			
		TP	FN	FP	TN	Acc.	Pre.	Recall	F1
1	Normal	497	19	16	480	96.542	96.881	96.774	96.828
2	DoS	364	17	16	615	96.739	95.789	97.464	96.620
3	Probe	87	4	6	915	99.012	93.548	99.349	96.361
4	R2L	12	1	1	998	99.801	92.308	99.899	95.954
5	U2R	11	0	2	999	99.802	84.615	99.800	91.583
**Summation**						491.897	463.142	493.287	477.345
**Average**						98.379	92.628	98.657	95.469

**Table 5:** Confusion matrix of NSL-KDD dataset.

**Actual class**	Normal	497	14	4	0	1	516
	DoS	14	364	2	1	0	381
	Probe	2	1	87	0	1	91
	R2L	0	1	0	12	0	13
	U2R	0	0	0	0	11	11
	**∑**	513	380	93	13	13	1012
		Normal	DoS	Probe	R2L	U2R	**∑**
		**Predicted class**					

**Fig 7:**
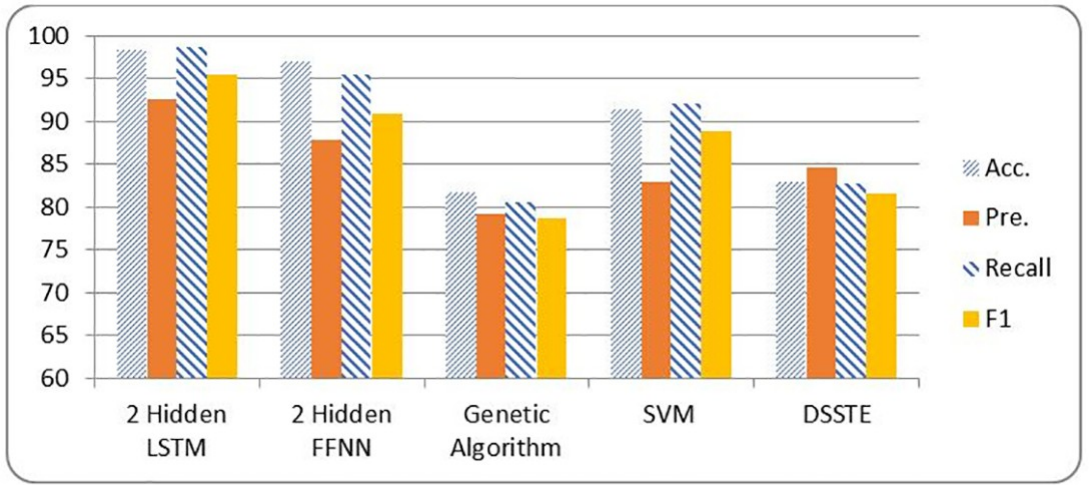
NSL-KDD metrics comparison among different classification techniques.

**Fig 8:**
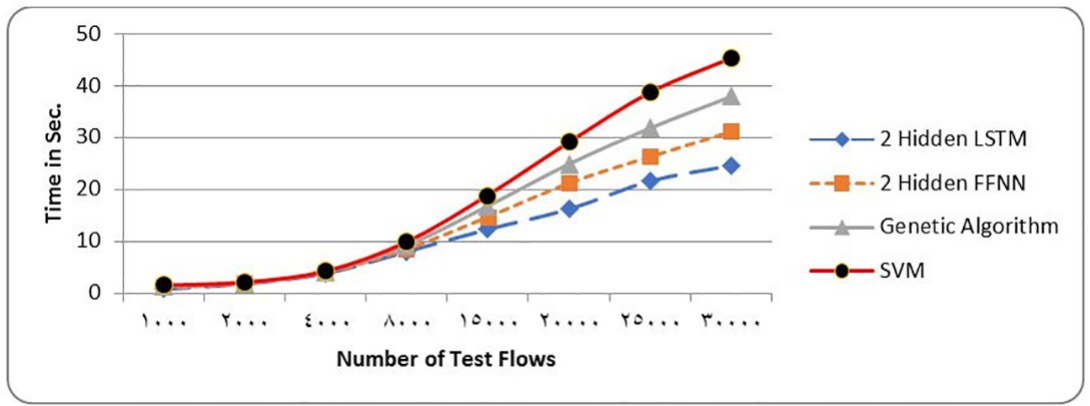
NSL-KDD Time comparison among different classification techniques.

Table 6 and Figs 7 and 8 show the metrics of a two layer LSTM-NN classification compared with a two layer FFNN, Genetic Algorithm (GA), Support Vector Machine (SVM) and Difficult Set Sampling Technique (DSSTE) algorithm. The DSSTE algorithm employs both Edited Nearest Neighbor (ENN) and K-Means clustering algorithms to reduce the data set’s majority class for improving the classifier’s training stage consequently enhances performance. The results show, using two hidden layers LSTM-NN provides best performance and time.

**Table 6:** NSL-KDD Comparison results among different classifiers.

Classification algorithm	NSL-KDD (%)				
	Acc.	Pre.	Recall	F1	Time(S)
2 Hidden LSTM	**98.379**	**92.628**	**98.657**	**95.469**	**11**
2 Hidden FFNN	97.017	87.912	95.481	90.913	15
Genetic Algorithm	81.764	79.184	80.541	78.683	21
SVM	91.415	82.878	92.104	88.799	26
DSSTE [45]	82.840	84.680	82.780	81.660	-

**Experiment 2**: (CIC-IDS2018 dataset): using a two hidden layers tuned LSTM-NN classifier.

Table 7 show the advanced metrics obtained from the confusion matrix in Table 8. The average results are 99.849, 91.536, 99.062 and 94.819 for Accuracy, Precision, Recall and F1 Score, respectively.

**Table 7:** CIC-IDS2018 metrics of the above confusion matrix.

No.	New Labels	Confusion matrix metrics				Advanced evaluation metrics (%)			
		TP	FN	FP	TN	Acc.	Pre.	Recall	F1
1	Normal	588,462	1,310	1,603	116,276	99.588	99.728	99.778	99.753
2	Botnet ARES	490	1	33	707,109	99.995	93.690	99.796	96.647
3	Brute Force	3,431	28	198	703,974	99.968	94.544	99.191	96.812
4	DoS/DDoS	72,247	1,379	1,141	632,859	99.644	98.445	98.127	98.286
5	Infiltration	9	0	4	707,623	99.999	69.231	100	81.818
6	PortScan	38,722	1,010	672	667,221	99.762	98.294	97.458	97.874
7	Web Attack	540	5	82	707,009	99.988	86.817	99.083	92.545
**Summation**						698.945	640.749	693.432	663.735
**Average**						99.849	91.536	99.062	94.819

**Table 8:** Confusion matrix of CIC-IDS2018 dataset.

**Actual class**	Normal	588,452	24	99	829	0	352	16	589,772
	Botnet ARES	2	485	1	0	2	0	1	491
	Brute Force	12	0	3,421	1	1	0	24	3,459
	DoS/DDoS	994	8	44	72,227	0	335	18	73,626
	Infiltration	0	0	0	0	9	0	0	9
	Port Scan	604	3	64	329	1	38,702	29	39,732
	Web Attack	1	3	0	2	0	5	534	545
	**∑**	590,065	523	3,629	73,388	13	39,394	622	707,634
		Normal	Botnet ARES	Brute Force	DoS/ DDoS	Infiltra-tion	Port Scan	Web Attack	**∑**
		**Predicted class**							

**Fig 9:**
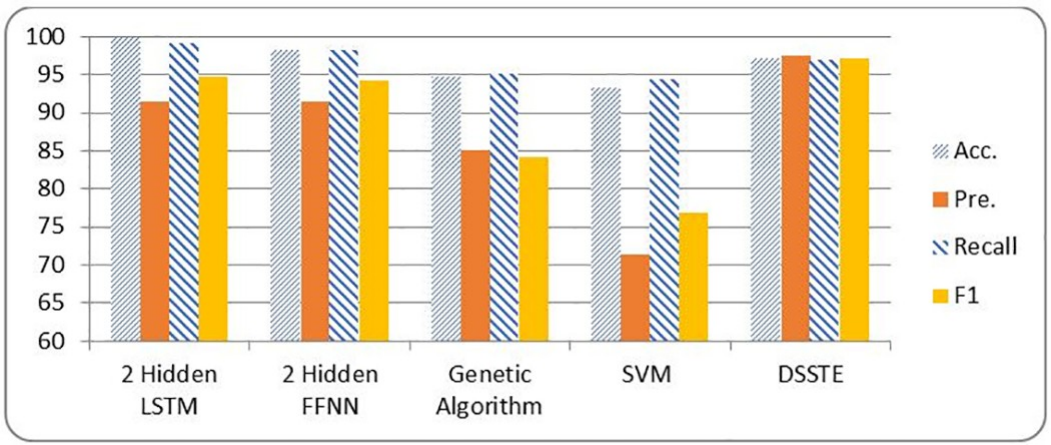
CIC-IDS2018 metrics comparison among different classification techniques.

**Fig 10:**
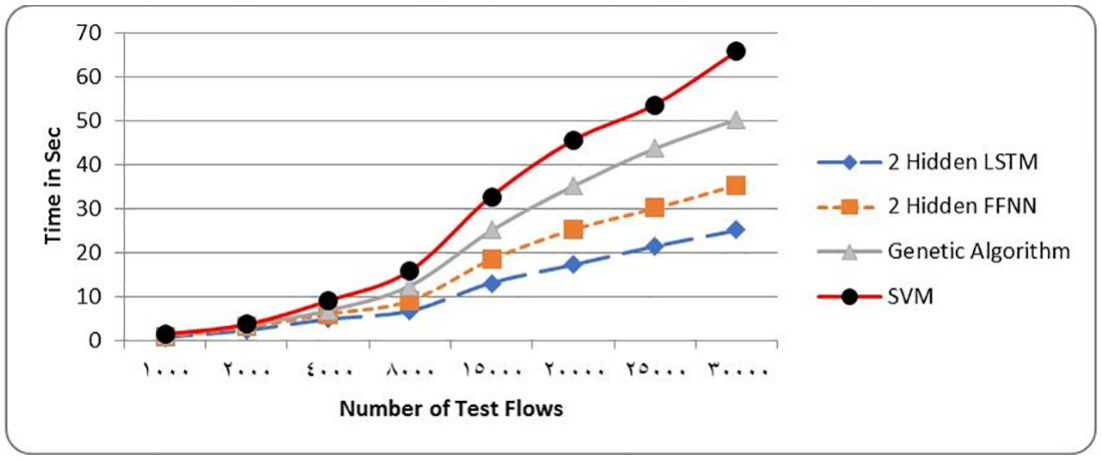
CIC-IDS Time comparison among different classification techniques.

As shown in Table 9 and Figs 9 and 10, the LSTM-NN provides good performance and running time.

**Table 9:** CIC-IDS2018 Comparison results among different classifiers.

Classification algorithm	CIC-IDS2018 (%)				
	Acc.	Pre.	Recall	F1	Time(S)
2 Hidden LSTM	**99.849**	91.536	**99.062**	94.819	**14**
2 Hidden FFNN	98.148	91.392	98.194	94.257	19
Genetic Algorithm	94.703	85.114	95.147	84.172	27
SVM	93.154	71.458	94.345	76.845	36
DSSTE [45]	96.990	**97.460**	96.970	**97.040**	-

In the next experiment, the proposed framework is evaluated using SDN dataset collected from the Mininet emulator and comparing its results with those of the framework introduced in [46].

**Experiment 3**: (SDN dataset): deploying a two hidden layers LSTM-NN classifier.

Table 10 shows the average performance metrics (Accuracy, Precision, Recall and F1 Score) obtained from confusion matrix in Table 11.

**Table 10:** SDN metrics of the above confusion matrix.

No.	Traffic Labels	Confusion matrix metrics				Advanced evaluation metrics (%)			
		TP	FN	FP	TN	Acc.	Pre.	Recall	F1
1	Benign ICMP	24,652	295	423	77,950	99.305	98.313	98.817	98.565
2	Malicious ICMP	15,775	239	252	87,054	99.525	98.428	98.508	98.468
3	Benign TCP	18,505	317	290	84,208	99.413	98.457	98.316	98.386
4	Malicious TCP	10,264	205	127	92,724	99.679	98.778	98.042	98.408
5	Benign UDP	22,159	393	358	80,410	99.273	98.410	98.257	98.334
6	Malicious UDP	10,375	141	140	92,664	99.728	98.669	98.659	98.664
**Summation**						596.922	591.054	590.599	590.825
**Average**						99.487	98.509	98.433	98.471

**Table 11:** Confusion matrix of SDN dataset.

**Actual class**	Benign ICMP	24,652	58	76	32	101	28	24,947
	Malicious ICMP	79	15,775	51	20	68	21	16,014
	Benign TCP	109	56	18,505	30	94	28	18,822
	Malicious TCP	57	35	42	10,264	53	18	10,469
	Benign UDP	129	82	93	43	22,159	46	22,552
	Malicious UDP	49	21	28	2	42	10,374	10,516
	**∑**	25,075	16,027	18,795	10,391	22,517	10,515	103,320
		Benign ICMP	Malicious ICMP	Benign TCP	Malicious TCP	Benign UDP	Malicious UDP	**∑**
		**Predicted class**						

**Fig 11:**
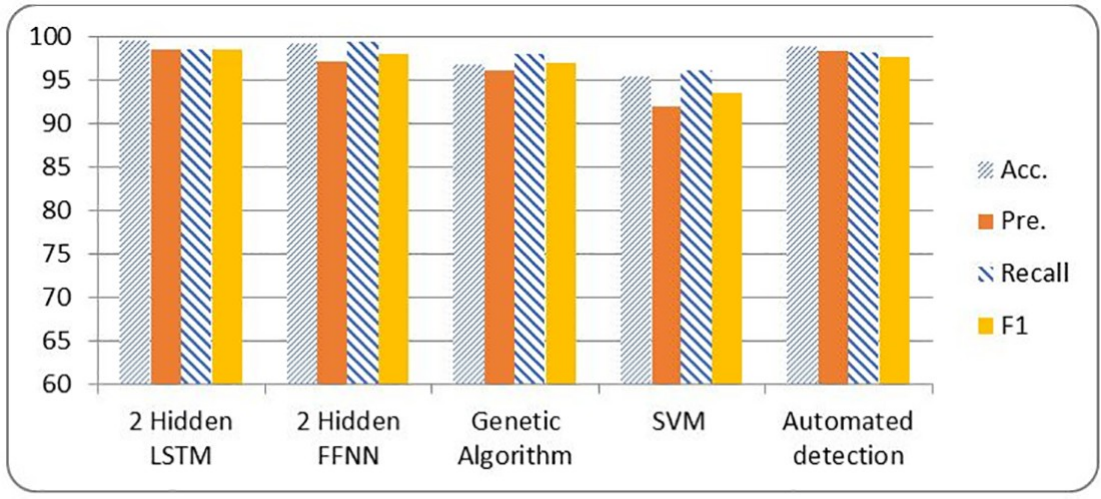
SDN metrics comparison among different classification techniques.

Table 12 and Fig 11 show a comparison in case of LSTM, FFNN, GA, SVM and Automated DDoS attack detection in SDN framework.

**Table 12:** SDN Comparison results among different classifiers.

Classification algorithm	NSL-KDD			
	Acc.	Pre.	Recall	F1
2 Hidden LSTM	**99.487**	**98.509**	**98.433**	**98.471**
2 Hidden FFNN	99.121	97.098	99.283	97.951
Genetic Algorithm	96.758	96.152	97.948	96.873
SVM	95.271	91.983	95.985	93.475
Automated detection [46]	98.800	98.270	98.180	97.650

## 6 Conclusions and future work

A Hybrid Deep Learning Intrusion Prediction IoT (HDLIP-IoT) Framework has been proposed in this paper as a tool for detection of DDoS attacks, to improve performance and minimize time of detection. It deploys both a signature-based and deep learning approach. An attack signature-based detection employs a list of previously caught attacks to detect threats in real time. The MWOA-LSTM approach is combined with the MWOA feature extraction in the deep learning layer. Primarily, it uses MWOA to extract feature from IP packets, and MWOA-LSTM is established as a method for predicting network traffic to identify DDoS attacks. Here, MWOA has been employed to select the best weight for NN hence the classification and prediction are as accurate as possible. Likewise, to make the best classification of attacks, the LSTM has been chosen for its ability to retain memory for a long period of time. To assess the superiority of the proposed framework, experiments are conducted on several datasets. Additionally, it identifies DDoS attacks with high accuracy and low latency to avoid adverse effects caused by DDoS attacks in IoT-SDN environments.

## Supporting information

S1 File Model explain. (ZIP)

S2 File IoT datasets files. (ZIP)

S3 File NSL-KDD dataset file. (ZIP)

S4 File Coding files. (ZIP)

S5 File O/P files. (ZIP)
