# Supplementary figures and images for: The proposed hybrid deep learning intrusion prediction IoT (HDLIP-IoT) framework
Source: PLoS One. 2022 Jul 29;17(7):e0271436. doi: 10.1371/journal.pone.0271436 (PMC9337696; doi:10.1371/journal.pone.0271436)

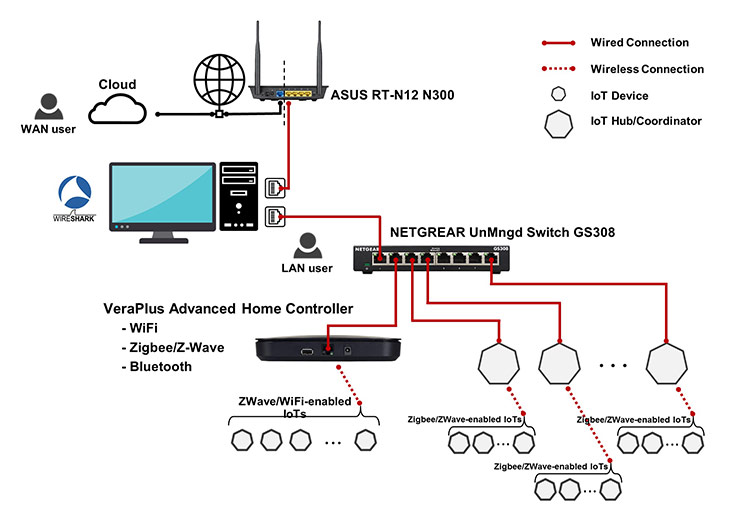

Supplement: S1 File — (ZIP) [file pone.0271436.s001.zip › iot-dataset.jpg]

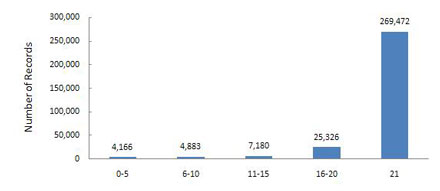

Supplement: S3 File — (ZIP) [file pone.0271436.s003.zip › KDDTest1.jpg]

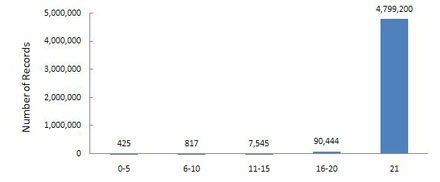

Supplement: S3 File — (ZIP) [file pone.0271436.s003.zip › KDDTrain1.jpg]
