# Supplementary material for: The proposed hybrid deep learning intrusion prediction IoT (HDLIP-IoT) framework
Source: PLoS One. 2022 Jul 29;17(7):e0271436. doi: 10.1371/journal.pone.0271436 (PMC9337696; doi:10.1371/journal.pone.0271436)
Supplement: S3 File — (ZIP) [file pone.0271436.s003.zip › index.html]

The NSL-KDD Data Set


|
|  |
| The NSL-KDD Data Set | |
|
| Abstract | |
|
| NSL-KDD is a data set suggested to solve some of the inherent problems of the KDD'99 data set which are mentioned in [1]. Although, this new version of the KDD data set still suffers from some of the problems discussed by McHugh [2] and may not be a perfect representative of existing real networks, because of the lack of public data sets for network-based IDSs, we believe it still can be applied as an effective benchmark data set to help researchers compare different intrusion detection methods. Furthermore, the number of records in the NSL-KDD train and test sets are reasonable. This advantage makes it affordable to run the experiments on the complete set without the need to randomly select a small portion. Consequently, evaluation results of different research work will be consistent and comparable. | |
| Data Files | ||
|
| KDDTrain+.ARFF | The full NSL-KDD train set with binary labels in ARFF format |
| KDDTrain+.TXT | The full NSL-KDD train set including attack-type labels and difficulty level in CSV format |
| KDDTrain+\_20Percent.ARFF | A 20% subset of the KDDTrain+.arff file |
| KDDTrain+\_20Percent.TXT | A 20% subset of the KDDTrain+.txt file |
| KDDTest+.ARFF | The full NSL-KDD test set with binary labels in ARFF format |
| KDDTest+.TXT | The full NSL-KDD test set including attack-type labels and difficulty level in CSV format |
| KDDTest-21.ARFF | A subset of the KDDTest+.arff file which does not include records with difficulty level of 21 out of 21 |
| KDDTest-21.TXT | A subset of the KDDTest+.txt file which does not include records with difficulty level of 21 out of 21 |
  | || Improvements to the KDD'99 data set | |
|
| The NSL-KDD data set has the following advantages over the original KDD data set:  - It does not include redundant records in the train   set, so the classifiers will not be biased towards more frequent records.  - There is no duplicate records in the proposed test   sets; therefore, the performance of the learners are not biased by the methods which   have better detection rates on the frequent records.  - The number of selected records from each difficultylevel   group is inversely proportional to the percentage of records in the original KDD   data set. As a result, the classification rates of distinct machine learning methods   vary in a wider range, which makes it more efficient to have an accurate evaluation   of different learning techniques.  - The number of records in the train and test sets   are reasonable, which makes it affordable to run the experiments on the complete   set without the need to randomly select a small portion. Consequently, evaluation   results of different research works will be consistent and comparable. | |
| Statistical Observations | |
|
| One of the most important deficiencies in the KDD data set is the huge number of redundant records, which causes the learning algorithms to be biased towards the frequent records, and thus prevent them from learning unfrequent records which are usually more harmful to networks such as U2R and R2L attacks. In addition, the existence of these repeated records in the test set will cause the evaluation results to be biased by the methods which have better detection rates on the frequent records. | |

|
|  |
|  | |  |  |  |  | | --- | --- | --- | --- | |  | Original Records | Distinct Records | Reduction Rate | | Attacks | 3,925,650 | 262,178 | 93.32% | | Normal | 972,781 | 812,814 | 16.44% | | Total | 4,898,431 | 1,074,992 | 78.05% | |  | |  |  |  |  | | --- | --- | --- | --- | |  | Original Records | Distinct Records | Reduction Rate | | Attacks | 250,436 | 29,378 | 88.26% | | Normal | 60,591 | 47,911 | 20.92% | | Total | 311,027 | 77,289 | 75.15% | |
|  | Statistics of redundant records in the KDD train set |  | Statistics of redundant records in the KDD test set |

|
|  |
| In addition, we analyzed the difficulty level of the records in KDD data set. Surprisingly, about 98% of the records in the train set and 86% of the records in the test set were correctly classified with all the 21 learners.   In order to perform our experiments, we randomly created three smaller subsets of the KDD train set each of which included fifty thousand records of information. Each of the learners where trained over the created train sets. We then employed the 21 learned machines (7 learners, each trained 3 times) to label the records of the entire KDD train and test sets, which provides us with 21 predicated labels for each record. Further, we annotated each record of the data set with a *#successfulPrediction* value, which was initialized to zero. Now, since the KDD data set provides the correct label for each record, we compared the predicated label of each record given by a specific learner with the actual label, where we incremented *#successfulPrediction* by one if a match was found. Through this process, we calculated the number of learners that were able to correctly label that given record. The highest value for *#successfulPrediction* is 21, which conveys the fact that all learners were able to correctly predict the label of that record. | | | |
|
|  |  |  |  |
|  | The distribution of *#successfulPrediction* values for the KDD train set records |  | The distribution of *#successfulPrediction* values for the KDD test set records |

|
|  |
| Refrences | |
|
| [1] M. Tavallaee, E. Bagheri, W. Lu, and A. Ghorbani, �A Detailed Analysis of the KDD CUP 99 Data Set,� *Submitted to Second IEEE Symposium on Computational Intelligence for Security and Defense Applications (CISDA)*, 2009.     [2] J. McHugh, �Testing intrusion detection systems: a critique of the 1998 and 1999 darpa intrusion detection system evaluations as performed by lincoln laboratory,� *ACM Transactions on Information and System Security*, vol. 3, no. 4, pp. 262�294, 2000. |
|
